# Instrumental Assessment of Stepping in Place Captures Clinically Relevant Motor Symptoms of Parkinson’s Disease

**DOI:** 10.3390/s20195465

**Published:** 2020-09-23

**Authors:** Karen Otte, Tobias Ellermeyer, Tim-Sebastian Vater, Marlen Voigt, Daniel Kroneberg, Ludwig Rasche, Theresa Krüger, Hanna Maria Röhling, Bastian Kayser, Sebastian Mansow-Model, Fabian Klostermann, Alexander Ulrich Brandt, Friedemann Paul, Axel Lipp, Tanja Schmitz-Hübsch

**Affiliations:** 1 NeuroCure Clinical Research Center, Charité–Universitätsmedizin Berlin, Corporate Member of Freie Universität Berlin, Humboldt-Universität zu Berlin, and Berlin Institute of Health, 10117 Berlin, Germany; tesakrueger@win.tu-berlin.de (T.K.); hanna.roehling@motognosis.com (H.M.R.); alexander.brandt@charite.de (A.U.B.); friedemann.paul@charite.de (F.P.); 2 Motognosis GmbH, 10119 Berlin, Germany; bastian.kayser@motognosis.com (B.K.); smm@motognosis.com (S.M.-M.); 3 Movement Disorders and Neuromodulation Unit, Department of Neurology, Charité–Universitätsmedizin Berlin, Corporate Member of Freie Universität Berlin, Humboldt-Universität zu Berlin, and Berlin Institute of Health, 10117 Berlin, Germany; tobiasellermeyer@web.de (T.E.); tim-sebastian.vater@charite.de (T.-S.V.); marlen.voigt@charite.de (M.V.); daniel.kroneberg@charite.de (D.K.); fabian.klostermann@charite.de (F.K.); lipp@park-klinik.com (A.L.); 4 Department of Neurology, Vivantes Auguste-Viktoria-Klinikum, 12157 Berlin, Germany; 5 Experimental and Clinical Research Center, Charité-Universitätsmedizin Berlin Corporate Member of Freie Universität Berlin, Humboldt-Universität zu Berlin, and Berlin Institute of Health and Max Delbrück Center for Molecular Medicine, 13125 Berlin, Germany; ludwig.rasche@gmx.de; 6 Department of Neurology, Park-Klinik Weißensee, 13086 Berlin, Germany; 7 Department of Neurology, University of California, Irvine, CA 92868, USA; 8 Einstein Center for Neuroscience, 10117 Berlin, Germany

**Keywords:** RGBD camera, movement analysis, Parkinson’s disease, postural instability

## Abstract

Fluctuations of motor symptoms make clinical assessment in Parkinson’s disease a complex task. New technologies aim to quantify motor symptoms, and their remote application holds potential for a closer monitoring of treatment effects. The focus of this study was to explore the potential of a stepping in place task using RGB-Depth (RGBD) camera technology to assess motor symptoms of people with Parkinson’s disease. In total, 25 persons performed a 40 s stepping in place task in front of a single RGBD camera (Kinect for Xbox One) in up to two different therapeutic states. Eight kinematic parameters were derived from knee movements to describe features of hypokinesia, asymmetry, and arrhythmicity of stepping. To explore their potential clinical utility, these parameters were analyzed for their Spearman’s Rho rank correlation to clinical ratings, and for intraindividual changes between treatment conditions using standard response mean and paired *t*-test. Test performance not only differed between ON and OFF treatment conditions, but showed moderate correlations to clinical ratings, specifically ratings of postural instability (pull test). Furthermore, the test elicited freezing in some subjects. Results suggest that this single standardized motor task is a promising candidate to assess an array of relevant motor symptoms of Parkinson’s disease. The simple technical test setup would allow future use by patients themselves.

## 1. Introduction

Parkinson’s disease is a progressive neurodegenerative disease with peak of onset in the sixth decade of life. The brain structures and functions affected result in a movement disorder defined by specific motor dysfunctions [1]. Patients with Parkinson’s disease (PWPD) may suffer from different combinations of slowing and shortness of movement (bradykinesia), increased muscle tone (rigidity), tremor, and typical postural instability [1]. This results in a hypokinetic gait disturbance, which may also include freezing of gait (FOG), characterized by episodic hesitations of stepping, or inefficient stepping with high frequency (festination), resulting in episodic arrest of locomotor behavior [2,3,4]. FOG may be triggered by various factors, has been related to increased risk of falling, and is a hallmark of transition into advanced disease stages [2,5].

Today, several treatment options are available to relieve the symptoms of this disorder, and their appropriate dosing depends on close observation of motor signs [6]. Thus, the recognition of these motor signs is not only critical for the diagnosis of Parkinson’s disease (PD), but also forms the basis of treatment decisions in PWPD [7]. Several instruments are clinically used for this purpose: a standardized clinical rating scale, such as the ‘Movement Disorder Society—Unified Parkinson’s disease rating scale (MDS-UPDRS) [7] and the Hoehn and Yahr scale [8], or patient self-reported outcomes [9]. A general limitation when assessing PD motor symptoms in a clinical setting is their fluctuating nature. As a well-known and bothersome complication of long-term medication in PWPD, the presence and severity of symptoms may considerably change in the short-term, e.g., in relation to medication intake, which may span from rather unimpaired, in medication ON state, to immobile, when drug effects wear off (medication OFF). Furthermore, additional motor features, such as dyskinesia, may indicate adverse effects of PD treatment [10]. Single point clinical assessments are not able to capture such fluctuations, and clinical rating scales may have limited sensitivity to quantify small-range changes in motor symptoms.

A manifold of technologies is available for a potentially more sensitive and rater-independent quantification of motor functions [11], which previous reviews explored for their application in PD [12,13,14,15]. For the assessment of motor fluctuations in PWPD, two approaches have been proposed: (1) non-standardized assessment, i.e., continuous tracking during everyday activities, which requires wearable sensors [16,17]; and (2) multi-point assessment of relevant motor symptoms in standard motor tasks, which requires technologies that are easily applicable by patients themselves. In this study, we follow the second approach, using 3D full body motion capture by RGB-Depth (RGBD) consumer cameras. The technology has already been applied for movement analysis in the clinical context, e.g., to analyze postural control and gait in different neurological disorders (e.g., PD [18], multiple sclerosis [19], and ataxia [20]), and showed good agreement with marker-based motion analysis standards [21]. The analyses presented here are based on observations from lab-based assessments, but the simple application of this technology would allow future application as patient-based assessment.

The stepping in place task (SIP), where patients are asked to repetitively walk on a spot while suppressing forward locomotion, was used here, based on prior evidence and low requirement of recording space. The task is long known as a clinical test for vestibular dysfunction when performed with eyes closed [22], but may also test components of gait and postural control when performed with eyes open [23]. SIP was used by Nantel et al. [24] to analyze temporal parameters, such as cadence, time symmetry, and arrhythmicity, in PWPD with and without FOG, contrasting their performance to a group of healthy subjects. They were the first to report that SIP triggered a freezing of stepping movements in patients with FOG, which was later confirmed by Dijsseldonk et al. [25]. Based on these findings, an array of relevant PD-specific motor symptoms may potentially be assessed in stepping in place behavior.

Our study is the first to utilize RGBD technology to derive kinematic parameters from SIP, and our spatial analysis of stepping behavior extends previous descriptions of SIP performance in PWPD. The objective was to quantitatively describe PWPDs’ performance of an SIP motor assessment using data from a single RGBD camera. For this purpose, algorithms for kinematic parameter extraction were developed and outcomes were analyzed regarding their potential for clinical use. According to previous experience and published evidence, we expected the test to be feasible for most patients with PD, but might be experienced as challenging for those in higher disease stages. We further expected a relation between test performance and disease severity, which would be expressed in correlations of kinematic parameters to clinical ratings of disease severity, as well as parameter differences between recordings taken in OFF and ON conditions.

## 2. Materials and Methods

### 2.1. Subjects and Clinical Testing

In total, data from 25 PWPD were used in this work (see Table 1), originating from two studies performed at an academic medical center (Charité—Universitätsmedizin Berlin, Germany, IRB approval EA1/012/17 and EA1/216/15). The studies explored motor outcomes from an RGBD sensor as a secondary aim while measuring patients in different therapeutic states defined as ON (depending on study defined as either optimized deep brain stimulation (DBS) or optimized symptomatic medication) or OFF (defined as either standardized withdrawal of DBS or medication). Inclusion criteria for the two studies were the clinical diagnosis of PD, according to UK Brain Bank Criteria [26]. Patients with limitations in motor performance unrelated to PD, including major psychiatric or cognitive disturbance, were excluded. For the purpose of this analysis, we additionally excluded recordings with dyskinesia reported at the time of assessment. All participants gave written informed consent for the assessment, analysis, and scientific publication of findings. Study data can be made available only on reasonable request.

The dataset comprised 20 assessments in ON and 13 assessments in OFF (including 10 intraindividual data pairs of ON and OFF assessments). The sample size requirements were based on recommendations from [14] for technical feasibility studies, which suggests first trials in up to 10 participants. Each assessment consisted of the performance of the full MDS-UPDRS III, as well as one recording of the stepping in place task. From the MDS-UPDRS III, the total score (range 0–142) and ratings for freezing of gait (item 11, range 0–4) and pull test (item 12, range 0–4) were available for analysis.

### 2.2. Stepping in Place

The study protocol of both studies included a standardized instruction of SIP to induce performance in self-selected, comfortable pace. To avoid exhaustion after performance, the SIP was limited to 40 s recording length, starting from onset of performance to automated stop of recording. Participants were explicitly told to avoid forward movement, but received no further instructions on leg or arm movements, nor prior demonstration of the task. A short testing performance of the task was explicitly allowed. If participants moved further than 1 m forward, the task was repeated, while reminding them to remain on spot and avoid moving forward. The tests were performed in common street clothing and usual footwear, excluding heeled shoes. Very loose clothing was asked to be taken off.

### 2.3. Technical Setup

**Figure 1:**
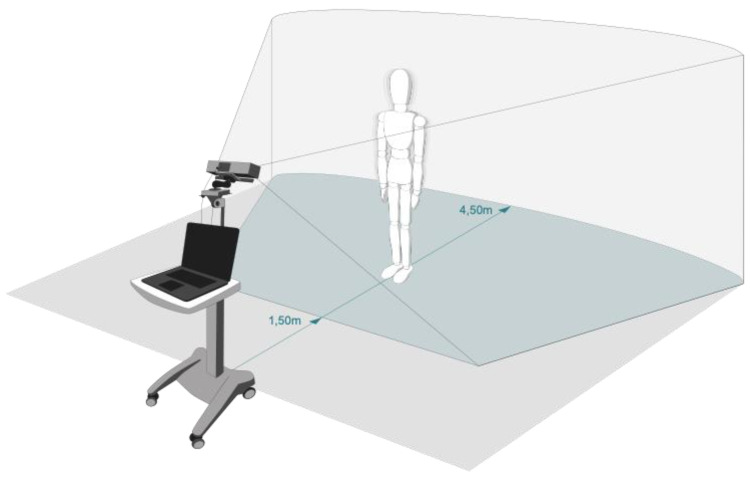
Technical setup of the motion capture system. Kinect camera was attached at 1.4 m height on a movable trolley with a pitch angle of roughly −9° while participants stood at a 2.5 m distance.

Instrumental recording of SIP used a marker-free motion capture technology based on a consumer RGBD camera (Microsoft Kinect for Xbox One). The Kinect camera was accessed by the official Microsoft Kinect SDK (Version 14.09) at a framerate of 30 Hz, using software developed for that purpose (Motognosis Labs V1.2, Motognosis GmbH, Berlin, Germany). The camera was placed on a movable trolley at 1.4 m height with a vertical angle of −9° (see Figure 1). Since the area of highest depth resolution is between 1.5 and 3.5 m, participants were placed facing the camera at a 2.5 m distance.

**Figure 2:**
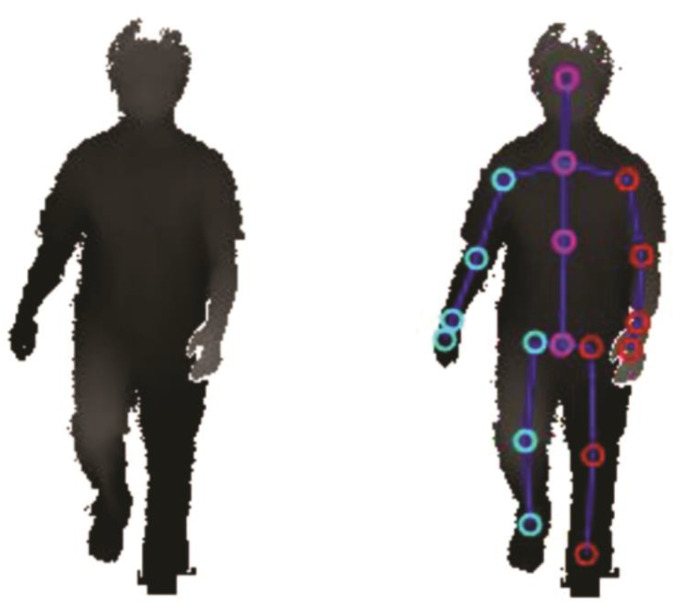
Recorded depth data of a masked participant (**left**) with 25 artificial anatomical landmarks (**right**) provided by the Kinect SDK.

The Microsoft Kinect SDK provided depth point clouds of the person in the measurement area, and identified 25 artificial anatomical landmarks (see Figure 2) representing the location of body parts and major joints (e.g., knees, ankles, hands, head), which were recorded and exported as .csv files.

### 2.4. Data Processing and Calculation of Kinematic Parameters

**Figure 3:**
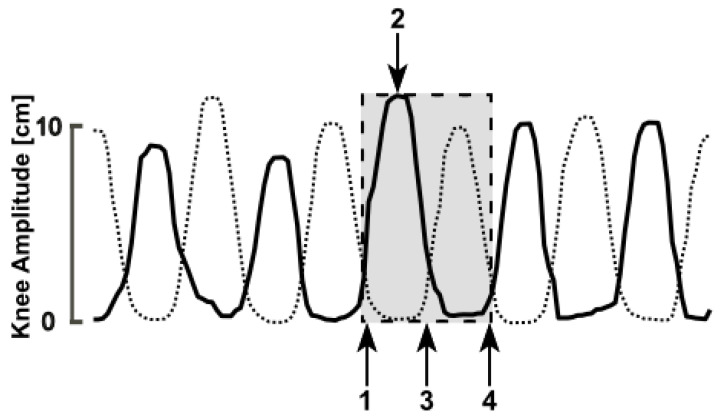
Representation of anterior–posterior movements of the right (thick black line) and left (thin dotted line) knee. The dashed box shows a complete stepping cycle including one step of each side. The following two phases are differentiated: Step phase of the right leg (from point 1 to 3) and stance phase (from point 3 to 4). Point 2 indicates the moment of anterior knee excursion (maximum hip flexion) and is used for the calculation of the knee amplitudes.

Since the anatomical landmarks of the feet and ankles tend to show noisy behavior during SIP according to prior technical validation [21], 3D knee movements were used to detect stepping behavior and to derive a parameter set for use in PWPD. Stepping movements were split in stance and step phases, similar to the stance and swing phase of each leg in a gait cycle during normal walking (see Figure 3).

Data pre-processing comprised the following steps:

1. To compensate for the subject’s position changes in the measurement area, we used the 3D positions of each knee as time series in relative position to the relating hip position. This eliminates possible errors due to the tendency to move towards the sensor.
2. A median filter (window size 5 frames) was applied to smoothen the anterior–posterior knee movement signal and reduce noise.
3. All minima of the filtered signal were detected and interpolated linearly, creating a minima-signal to provide a base level of minor landmark shifts over time caused by changes in the detected 3D user mask.
4. The minima-signal was subtracted from the anterior–posterior knee movement signal to eliminate smaller measurement errors when the knees were straight.
5. A threshold of 2.5 cm for anterior–posterior knee amplitude was defined as suitable to differentiate between step (>2.5 cm) and stance (<2.5 cm) phase. The threshold was identified by visual inspection of recordings.

From the detected step and stance phases, we derived eight kinematic parameters to describe major motor features of PD (Table 2). All parameters, besides cadence and asymmetry, were calculated separately for each body side, and then combined as their mean for further analysis.

Equations for the calculation of arrhythmicity (step time coefficient of variation) (1) and asymmetry (2) were taken from common definitions, as, for example, provided by Plotnik et al. [27].

(1) $$\mathrm{Arrhythmicity}=\;100*\left|\frac{\mathrm{std}\left(\mathrm{StepTimes}\right)}{\mathrm{mean}\left(\mathrm{StepTimes}\right)}\right|$$

(2) $$\mathrm{Asymmetry}=\;100*\left|\left(\frac{\mathrm{mean}({\mathrm{AmplitudesAmplitudes}}_{\mathrm{SmallerSide}}}{\mathrm{mean}\left({\mathrm{Amplitudes}}_{\mathrm{LargerSide}}\right)}\right)\right|$$

Since asymmetry is expressed as ratio between both sides, persons with small knee amplitudes show higher asymmetry measures for similar absolute amplitude differences.

### 2.5. Statistical Analysis

Descriptive statistics are given for metric kinematic parameters as mean and standard deviation. To explore confounding effects of age, height, and weight on SIP parameters, Pearson’s correlations were performed in the ON subgroup.

Relation to disease severity was explored by correlating pooled recordings with the corresponding MDS-UPDRS III total score and pull test score, using Spearman’s rank correlation. Pooled data is here used to provide higher heterogeneity in clinical symptom severity. From the subgroup of 10 patients with paired data from recordings in ON and OFF available, within-group comparisons were calculated between ON and OFF therapeutic states, reported as absolute and relative differences (percentage change from value in OFF condition), along with statistics from paired *t*-tests. Additionally, the standardized response mean (SRM) was provided as ratio of average difference and standard deviation of differences between OFF and ON. Due to the exploratory nature and small cohort size, analyses were not corrected for multiple comparisons, and the significance levels for all tests were set at 1%.

All statistics were calculated using Python 3.5 and the SciPy package version 0.18.1. Diagrams were created with Seaborn (package version 0.7.1) and Matplotlib (package version 2.0.0).

## 3. Results

### 3.1. Descriptive Statistics and Analysis of Potential Confounding Effects

The descriptive statistics of all eight derived kinematic parameters are provided for the pooled dataset, as well as the subsets of recordings acquired in ON and OFF (Table 3). In the subset of ON recordings, i.e., in a state of least expression of PD motor symptoms, the correlations between kinematic parameters and age, height, or body weight did not indicate relevant confounding effects by overall non-significant and small correlation coefficients (|*r*| < 0.27 for age, <0.15 for height and <0.36 for weight).

### 3.2. Relation of SIP Parameters to Disease Severity and Postural Instability

In total, two out of the eight parameters—knee amplitude and longest stance time—were correlated with clinical ratings MDS-UPDRS III, and another two (arrhythmicity and average stance time) showed a trend (*p* < 0.05). Specifically, knee amplitude was reduced in subjects with higher clinical ratings (MDS-UPDRS III rho = −0.507, *p*-value = 0.003), while longest stance time increased (rho = 0.523, *p*-value = 0.002). The correlations with pull test ratings of postural instability were in the same direction, but reached significance only for longest stance time (Table 4). Trends indicated an increase of arrhythmicity and average stance time with more severe clinical ratings.

### 3.3. Comparison between Recordings Taken in ON vs. OFF States

**Figure 4:**
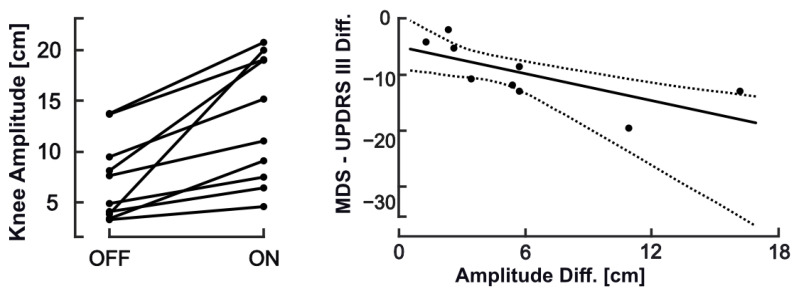
Changes of knee amplitude between treatment states (left) and knee amplitude changes related to disease severity change.

As expected, the clinical rating indicated relevant within-group change in motor symptoms from OFF to ON states (29% decrease in MDS-UPDRS III) in the subset with assessments available from both conditions. Changes in SIP behavior from OFF to ON were reflected in increase of knee amplitude (85.4%, *p*-value = 0.002) and average step time (14.5%, *p*-value = 0.007), a decrease of step asymmetry (−19.6%, *p*-value = 0.007) with a similar trend for arrhythmicity, while cadence remained unchanged (Table 5). On inspection of corresponding data plots, a consistent change between OFF and ON was only seen for knee amplitude, which closely reflected respective differences in MDS-UPDRS III (Figure 4). The pronounced increase in longest stance time from OFF to ON, though non-significant, was unexpected in direction. This parameter reflects hesitations in stepping that would be expected to become less with effective therapy. However, inspection of data revealed one very long FOG episode (>20 s) during one recording in ON condition with a relevant impact on parameter mean.

### 3.4. Implications of FOG and Other Motor Patterns

**Figure 5:**
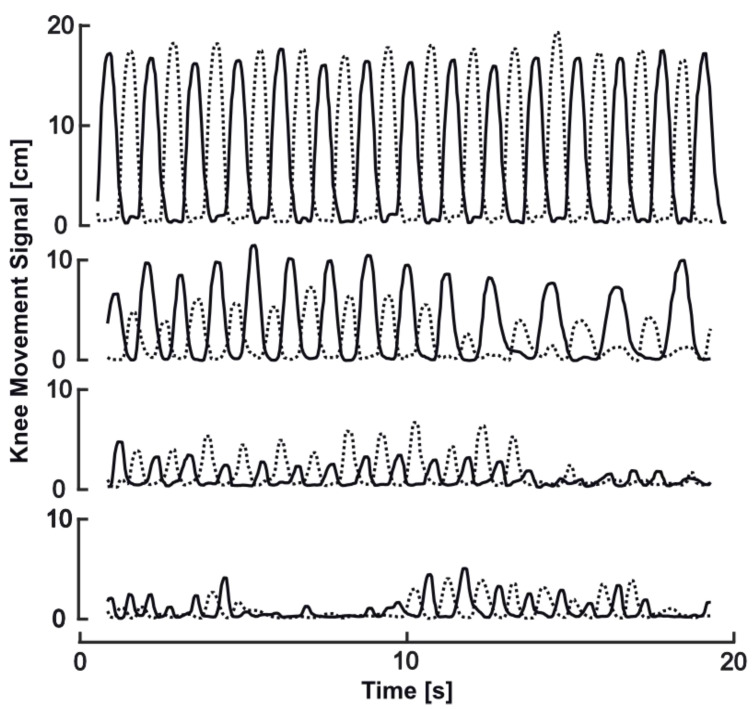
Illustration of freezing of gait (FOG) related changes on SIP behavior from our sample: normal rhythmic stepping behavior of regular and symmetric amplitude (**top**); hesitations of stepping with asymmetrical inconsistent stepping behavior and slowing of movements at the end (**second from top**); progressively ineffective stepping with small movement, asymmetry, and freezing of stepping at the end (**third from top**); ineffective highly irregular low-amplitude stepping and freezing of stepping in first half and at the end (**bottom**).

Freezing of stepping was clinically observed during 7 out of 33 recordings (ON: *n* = 6; OFF: *n* = 1). Examples from our sample (Figure 5) illustrate possible effects of FOG behavior on SIP parameters. In contrast to normal stepping behavior with constant rhythm and amplitude (Figure 5 top), hesitations and slower movements would be expected to result in increase of longest and average stance time, step time, and, therefore, lower cadence (Figure 5, line two from top). Related to this movement behavior, arrhythmicity can be found as well, and may show remarkable asymmetry (lines two and three). Festination prior to freezing manifestation may result in decrease of average knee amplitude, step, and stance timing, with remarkable asymmetry (third example). The manifestation of freezing will clearly result in massive increases of longest stance time as the prominent and possibly defining feature (lines three and four), usually in company with reduced knee amplitude and increased arrhythmicity.

## 4. Discussion

Our study explored the instrumental assessment of motor signs in patients with Parkinson’s disease using SIP as a standard motor task performed in front of a single RGBD camera. Both technology and task were chosen for their potential application as patient-based assessment in the home setting, although recordings were done in the lab at this stage.

The 3D motion signals of knees were used to derive eight different kinematic parameters for the description of stepping behavior in SIP. This extends previous SIP descriptions [23,24,28] to the spatial domain, including amplitude and spatial symmetry of stepping. Although foot signals from RGBD recordings have been used for step detection in normal gait [29,30], we preferred knee signals, because they showed less noise behavior compared to foot and ankle landmarks in an earlier validation of our system [21]. The kinematic parameters were selected to reflect key motor aspects of PD. Knee amplitude and step time are conceived to describe hypo/bradykinesia, similar to shortening of stepping during gait at self-selected speed, which can be considered the main gait characteristic in PWPD [31,32]. Temporal asymmetry is an important feature, specifically in the early stages of the disease [33,34], and reduced interlimb coordination has also been related to FOG [4,27]. Interestingly, spatial asymmetry of stepping during gait has been related to postural control [35] instead of temporal asymmetry, and might be specifically affected in subtypes of PWPD [36].

Changes in cadence were not consistently seen in previous gait descriptions in PWPD, but an increase of cadence and shorter step times in PWPD may indicate festination of stepping. In contrast, increasing stance times may indicate hesitations, and excessive longest stance times may indicate episodes of ineffective stepping or freezing. Variability of stepping, specifically step and stride timing during gait, forms a separate domain of gait as conceptualized by Lord et al. [32], which has gained increasing interest in the assessment of PWPD [16,37]. We therefore included arrhythmicity of stepping, similar to the coefficient of variance for step or stride time that is used as common descriptor in gait analysis, which is sensitive to number of steps, as well as the gait paradigm used for recording [38]. Although the similarity of SIP movement to stepping during gait is intriguing, we are aware of only one small study [23] which compared cadence from SIP and gait recordings. Thus, our parameter wording should not imply that we consider specific parameters directly comparable to gait descriptions in PWPD. We therefore also refer to freezing of stepping in our observations, although it obviously shares features with FOG.

Prior to this work, there were only a few publications on the instrumental assessments of SIP. In our study, stepping in place was instructed to evoke self-selected stepping pace without any external cueing. Differences in task instructions as well as sample characteristics may contribute to explain the slightly lower cadence reported here, compared to previous reports (97–99 steps/min in our study vs. 100–112 steps/min from [23,24,28]). Derived spatial asymmetry values presented in this work were notably higher than the reported temporal swing time asymmetry during SIP by Nantel et al. [24], which may indicate limitations in the comparability of spatial and temporal asymmetry measures. Comparability of measurements to age-matched healthy volunteers should be considered for future works, to define normal stepping behavior in this task and corroborate evidence on analogies and differences to stepping behavior during gait. Furthermore, although our results did not suggest dependency on age, body height, or weight, potential confounders need to be analyzed in more appropriate datasets, as well as variability of performance with repeated testing. For use in PWPD, this test series showed excellent applicability of RGBD-instrumented SIP, even in higher disease stages. Still, the need for well-standardized procedures of data acquisition and for quality control of acquired data, specifically in remote application, needs not be neglected to make this a useful aid to clinicians and disease management in PWPD.

With respect to clinical validity, correlation analysis in our cohort indicated that smaller knee amplitude and longer stance times reflect higher disease severity. As knee amplitude can be conceived as the spatial parameter of stepping, this finding corresponds well to reduced step length during gait. While stance time during gait may increase with need to stabilize gait, often in parallel with reduction in gait speed, it has, to our knowledge, not been explored as an indicator of hesitations in stepping, nor has longest stance time been reported as an indicator of FOG episodes. Our observation of excessive longest stance time in individuals who experience freezing of stepping during SIP would support this concept. Future study may define useful thresholds for an automated detection of freezing and related behaviors, as exemplified in Figure 5. Both knee amplitude and longest stance time, but also arrhythmicity, showed substantial correlations to the clinical rating of postural instability from pull test performance. This is remarkable, as postural instability in PWPD is a motor feature of high clinical relevance regarding prognosis, fall risk, and interventions, yet hard to assess clinically. Pull test performance and rating notably suffers low reliability [39]. Therefore, future study should aim to corroborate this finding, which further supports the notion that SIP tests aspects of postural control, in addition to aspects of gait.

From the 33 SIP recordings, seven included freezing episodes, according to operator observation, as well as inspection of knee signals. This supports the notion that the SIP task triggers freezing of stepping [24,25]. The occurrence of FOG is known to depend on environmental cues as well as the type of motor task, where increased task complexity and cognitive demands increase FOG appearance [40,41]. Previous reports indicating cognitive demand of SIP execution [28] could explain the appearance of freezing in this task. However, our study was not designed to further explore the diagnostic accuracy of SIP for FOG detections. This would need a study design that compares matched samples of freezers and non-freezers, defined along established standards and against more detailed clinical ratings of FOG and related phenomena. Other motor tasks, such as 360° turns or walking through doorways, might have a higher probability of triggering FOG [25]. Unfortunately, due to occlusion of body parts during execution, the extraction of reliable kinematic parameters from turn tasks proves difficult when using markerless motion capture technology.

The comparison between ON and OFF recordings from a subgroup of 10 PWPD aimed to explore the sensitivity to effects of intervention. As expected, these were reflected in a decrease in MDS-UPDRS III from OFF to ON, which can be considered as clinically relevant, both with regard to absolute and relative change [42,43]. This overt change in clinical state, however, left cadence unchanged, while knee amplitude and average step time increased and spatial asymmetry decreased (trend for decrease in arrhythmicity). Specifically, the average proportional increase of knee amplitude (85%) was much higher than the relative change observed in clinical ratings (−29%), suggestive of a higher sensitivity to change compared to clinical rating. This can, however, only be confirmed if retest reliability has been determined. Concerning average step time, the appearance of a very long FOG episode (>20 s) in one ON recording might have influenced statistical analysis of this parameter, and explains the massive increase in longest stance time for OFF to ON.

The advantage of markerfree motion capture, in comparison to single or multiple wearable sensors, is the potential analysis of the full body. After consideration of required accuracy levels, signal analysis may extend to other body parts, and could be used to describe arm swing or torso sway dynamics during task performance. From clinical observation, such measures seem interesting candidates for a description of dyskinesia in PWPD. This was not considered in our study, as recordings with dyskinesia were deliberately excluded, but is clearly needed in further validation of this task for clinical application in PWPD.

From this first kinematic analysis, SIP alone may capture a variety of clinically relevant PD motor symptoms within one test, including FOG, although the relation of SIP performance to hand functions and dyskinesia have not yet been investigated. The task could also be modified with respect to duration or by adding cognitive or motor dual task conditions. The literature shows that, in conventional gait tasks, adding further cognitive load via dual tasking increases FOG appearance and alters movement patterns in PWPD [8,44]. Whether this also holds true for SIP, as a non-locomotor stepping task, still needs to be shown.

## 5. Conclusions

In this sample of 25 PWPD, all patients were able to perform the 40 s stepping in place task in OFF and ON therapeutic state. Freezing episodes were seen during some of the SIP performances in OFF as well as ON, confirming previous reports. From all recordings, a set of kinematic parameters were derived, describing range of movement (knee amplitude), arrhythmicity, and asymmetry, as well as stance timing. As an indicator of clinical validity, some parameters showed relations to clinical ratings of disease severity, specifically postural instability. Measures of knee amplitude showed also consistent changes between OFF and ON states, indicating high responsiveness of this parameter.

## Figures and Tables

**Table 1:** Description of subgroups, where metrical measures are given as mean and standard deviation and ordinal data as number of reached scores for each value.

	ALL	ON	OFF	ON-OFF
N subjects	25	20	13	10
male	18	15	8	6
female	7	5	5	4
Age (years)	65.3 (±9.4)	65.5 (±11.05)	66.2 (±8.0)	65.3 (±8.7)
Weight (kg)	75.0 (±13.5)	74.1 (±13.5)	76.3 (±12.5)	76.2 (±13.8)
Height (cm)	168.4 (±6.8)	167.7 (±6.1)	170.4 (±7.8)	168.4 (±5.3)
Disease Duration (years)	12.8 (±8.1)	12.1 (±8.0)	11.6 (±6.6)	10.1 (±7.2)
MDS-UPDRS-III	28.3 (±14.7)	25.3 (±13.7)	34.9 (±15.1)	ON: 28.8 (±13.4)
				OFF: 37.2 (±14.5)
N-item 11 (FOG)	23/4/6/0/0	14/3/3/0/0	9/1/3/0/0	ON: 8/2/0/0/0
0/1/2/3/4				OFF: 7/3/0/0/0
N-item 12 (Pull test)	12/10/6/1/1	8/4/5/2/0	4/6/1/1/1	ON: 4/3/2/1/0
0/1/2/3/4				OFF: 1/6/1/1/1

**Table 2:** Description of eight kinematic parameters from VPC recordings of stepping in place (SIP) task performance.

Parameter Name	Unit	Description
Cadence	Steps/min	Steps per minute
Knee Amplitude	cm	Anterior–posterior range of motion of knees
Asymmetry	%	Logarithmic ratio between knee amplitudes of larger side to smaller side
Average Step Time	s	Average time required for a step during the measurement
Longest Step Time	s	Maximal time required for a step during the measurement
Arrhythmicity	%	Ratio between standard deviation and average of the step time
Average Stance Time	s	Average time between step movements
Longest Stance Time	s	Maximal time between step movements

**Table 3:** Descriptive statistics for SIP parameters on the left for the whole sample and subset of recordings in ON and OFF treatments states, and on the right, estimation of confounding effects of age, height, and weight in the subset of recordings in ON.

	Descriptive Statistics			Confounder Analysis in ON (*n* = 20)		
	Mean (SD)			Pearson’s Correlation Coefficient *r* (*p*-Value)		
	ALL (*n* = 33)	ON (*n* = 20)	OFF (*n* = 13)	Age	Height	Weight
Cadence (steps/min)	97.6 (27.3)	96.6 (27.1)	99.2 (28.6)	−0.271 (0.247)	0.132 (0.682)	−0.358 (0.253)
Knee Amplitude (cm)	12.5 (7.4)	13.9 (5.5)	10.2 (9.3)	0.228 (0.334)	0.151 (0.640)	0.280 (0.378)
Asymmetry (%)	18.2 (19.9)	15.6 (14.1)	22.1 (26.8)	−0.133 (0.577)	0.066 (0.839)	−0.173 (0.591)
Average Step Time (s)	0.72 (0.21)	0.77 (0.21)	0.64 (0.19)	−0.114 (0.632)	−0.108 (0.739)	−0.215 (0.503)
Longest Step Time (s)	0.88 (0.24)	0.93 (0.23)	0.80 (0.26)	0.209 (0.376)	−0.038 (0.906)	0.259 (0.417)
Arrhythmicity (%)	11.6 (5.58)	11.3 (6.5)	12.2 (4.0)	0.264 (0.261)	0.118 (0.715)	0.350 (0.265)
Average Stance Time (s)	0.65 (0.60)	0.61 (0.60)	0.72 (0.61)	0.258 (0.272)	0.108 (0.739)	0.348 (0.267)
Longest Stance Time (s)	1.69 (2.39)	1.63 (2.75)	1.77 (1.77)	−0.271 (0.247)	0.132 (0.682)	−0.358 (0.253)

**Table 4:** Spearman’s rank correlation of the eight kinematic parameters with clinical ratings acquired at the time of each SIP recording; analyzed from the pooled dataset (*n* = 33).

	Spear. Corr. MDS-UPDRS III	Spear. Corr. Pull Test
	Rho (*p*-Value)	Rho (*p*-Value)
Cadence (steps/min)	−0.234 (0.189)	−0.328 (0.072)
Knee Amplitude (cm)	−**0.507 (0.003)**	−0.436 (0.014) *
Asymmetry (%)	0.202 (0.260)	0.170 (0.361)
Average Step Time (s)	−0.287 (0.105)	−0.274 (0.136)
Longest Step Time (s)	−0.291 (0.101)	−0.242 (0.191)
Arrhythmicity (%)	0.352 (0.045) *	0.452 (0.011) *
Average Stance Time (s)	0.374 (0.032) *	0.374 (0.038) *
Longest Stance Time (s)	**0.523 (0.002)**	**0.468 (0.008)**

Statistically significant outcomes are set in bold; * indicates trend (*p*-value < 0.05).

**Table 5:** Changes in SIP parameters and clinical rating from OFF to ON state in the subset with assessments in both conditions available (*n* = 10).

	Mean (SD) OFF	Mean (SD) ON	Diff Abs.	Diff [%]	SRM	Paired *t*-Test *p*-Value
**MDS-UPDRS III**	**37.2 (14.53)**	**28.8 (13.37)**	**10.64**	**−28.6**	1.69	**<0.001**
Cadence (steps/min)	96.6 (29.0)	96.9 (20.7)	0.36	0.4	−0.02	0.954
**Knee Amplitude (cm)**	**7.08 (4.0)**	**13.1 (6.2)**	**6.05**	**85.4**	−1.34	**0.002**
**Asymmetry (%)**	**21.9 (27.6)**	**17.7 (18.3)**	**−4.30**	**−19.6**	0.14	**0.007**
**Average Step Time (s)**	**0.61 (0.17)**	**0.71 (0.14)**	**0.09**	**14.5**	−1.09	**0.007**
Longest Step Time (s)	0.76 (0.21)	0.81 (0.14)	0.05	7.2	−0.35	0.298
Arrhythmicity (%)	11.9 (3.50)	8.46 (3.93)	−3.49	−29.3	0.84	0.025
Average Stance Time (s)	0.80 (0.67)	0.62 (0.51)	−0.18	−23.0	0.55	0.114
Longest Stance Time (s)	1.88 (1.87)	3.67 (8.04)	1.79	94.6	−0.27	0.423

Statistically significant outcomes are set in bold.

## References

[1] Jankovic J. (2008). Parkinson’s disease: Clinical features and diagnosis. J. Neurol. Neurosurg. Psychiatry.

[2] Giladi N., Treves T.A., Simon E.S., Shabtai H., Orlov Y., Kandinov B., Paleacu D., Korczyn A.D. (2001). Freezing of gait in patients with advanced Parkinson’s disease. J. Neural Transm..

[3] Martens K.A.E., Lukasik E.L., Georgiades M.J., Gilat M., Hall J., Walton C.C., Lewis S.J. (2018). Predicting the onset of freezing of gait: A longitudinal study. Mov. Disord..

[4] Nieuwboer A., Rochester L., Herman T., Vandenberghe W., Emil G.E., Thomaes T., Giladi N. (2009). Reliability of the new freezing of gait questionnaire: Agreement between patients with Parkinson’s disease and their carers. Gait Posture.

[5] Mancini M., Bloem B.R., Horak F.B., Lewis S.J.G., Nieuwboer A., Nonnekes J. (2019). Clinical and methodological challenges for assessing freezing of gait: Future perspectives. Mov. Disord..

[6] Fox S.H., Katzenschlager R., Lim S.Y., Ravina B., Seppi K., Coelho M., Poewe W., Rascol O., Goetz C.G., Sampaio C. (2011). The Movement Disorder Society Evidence-Based Medicine Review Update: Treatments for the motor symptoms of Parkinson’s disease. Mov. Disord..

[7] Postuma R.B., Berg D. (2017). The New Diagnostic Criteria for Parkinson’s Disease.

[8] Hoehn M.M., Yahr M.D. (1967). Parkinsonism: Onset, progression, and mortality. Neurology.

[9] Martinez-Martin P., Jeukens-Visser M., Lyons K.E., Rodriguez-Blazquez C., Selai C., Siderowf A., Welsh M., Poewe W., Rascol O., Sampaio C. (2011). Health-related quality-of-life scales in Parkinson’s disease: Critique and recommendations. Mov. Disord..

[10] Jankovic J. (2005). Motor fluctuations and dyskinesias in Parkinson’s disease: Clinical manifestations. Mov. Disord..

[11] Buckley C., Alcock L., McArdle R., Rehman R.Z.U., Del Din S., Mazzà C., Yarnall A.J., Rochester L. (2019). The role of movement analysis in diagnosing and monitoring neurodegenerative conditions: Insights from gait and postural control. Brain Sci..

[12] Sánchez-Ferro Á., Elshehabi M., Godinho C., Salkovic D., Hobert M.A., Domingos J., van Uem J.M., Ferreira J.J., Maetzler W. (2016). New methods for the assessment of Parkinson’s disease (2005 to 2015): A systematic review. Mov. Disord..

[13] Espay A.J., Bonato P., Nahab F., Maetzler W., Dean J.M., Klucken J., Eskofier B.M., Merola A., Horak F., Lang A.E. (2016). Technology in Parkinson disease: Challenges and Opportunities on behalf of the MDS Taskforce on Technology HHS Public Access Author manuscript. Mov. Disord..

[14] Maetzler W., Klucken J., Horne M. (2016). A clinical view on the development of technology-based tools in managing Parkinson’s disease. Mov. Disord..

[15] Godinho C., Domingos J., Cunha G.V., Santos A.T., Fernandes R.M., Abreu D., Gonçalves N., Matthews H., Isaacs T., Duffen J. (2016). A systematic review of the characteristics and validity of monitoring technologies to assess Parkinson’s disease. J. Neuroeng. Rehabil..

[16] Del Din S., Godfrey A., Mazzà C., Lord S., Rochester L. (2016). Free-living monitoring of Parkinson’s disease: Lessons from the field. Mov. Disord..

[17] Maetzler W., Domingos J., Srulijes K., Ferreira J.J., Bloem B.R. (2013). Quantitative wearable sensors for objective assessment of Parkinson’s disease. Mov. Disord..

[18] Galna B., Jackson D., Schofield G., McNaney R., Webster M., Barry G., Mhiripiri D., Balaam M., Olivier P., Rochester L. (2014). Retraining function in people with Parkinson’s disease using the Microsoft kinect: Game design and pilot testing. J. Neuroeng. Rehabil..

[19] Behrens J., Pfüller C., Mansow-Model S., Otte K., Paul F., Brandt A.U. (2014). Using perceptive computing in multiple sclerosis—The Short Maximum Speed Walk test. J. Neuroeng. Rehabil..

[20] Ilg W., Schatton C., Schicks J., Giese M.A., Schöls L., Synofzik M. (2012). Video game-based coordinative training improves ataxia in children with degenerative ataxia. Neurology.

[21] Otte K., Kayser B., Mansow-Model S., Verrel J., Paul F., Brandt A.U., Schmitz-Hübsch T. (2016). Accuracy and reliability of the kinect version 2 for clinical measurement of motor function. PLoS ONE.

[22] Fukuda T. (1959). The stepping test. Acta Oto-Laryngol..

[23] Garcia R.K., Nelson A.J., Ling W., Van Olden C. (2001). Comparing stepping-in-place and gait ability in adults with and without hemiplegia. Arch. Phys. Med. Rehabil..

[24] Nantel J., de Solages C., Bronte-Stewart H. (2011). Repetitive stepping in place identifies and measures freezing episodes in subjects with Parkinson’s disease. Gait Posture.

[25] Van DIjsseldonk K., Wang Y., Van Wezel R., Bloem B.R., Nonnekes J. (2018). Provoking freezing of gait in clinical practice: Turning in place is more effective than stepping in place. J. Parkinson’s Dis..

[26] Gibb W.R., Lees A.J. (1988). The relevance of the Lewy body to the pathogenesis of idiopathic Parkinson’s disease. J. Neurol. Neurosurg. Psychiatry.

[27] Plotnik M., Giladi N., Balash Y., Peretz C., Hausdorff J.M. (2005). Is freezing of gait in Parkinson’s disease related to asymmetric motor function?. Ann. Neurol..

[28] Dalton C., Sciadas R., Nantel J. (2016). Executive function is necessary for the regulation of the stepping activity when stepping in place in older adults. Aging Clin. Exp. Res..

[29] Mentiplay B., Perraton L.G., Bower K.J., Pua Y.-H., McGaw R., Heywood S., Clark R.A. (2015). Gait assessment using the Microsoft Xbox One Kinect: Concurrent validity and inter-day reliability of spatiotemporal and kinematic variables. J. Biomech..

[30] Steinert A., Sattler I., Otte K., Röhling H., Mansow-Model S., Müller-Werdan U. (2020). Using new camera-based technologies for gait analysis in older adults in comparison to the established GAITrite system. Sensors.

[31] Morris R., Lord S., Lawson R.A., Coleman S., Galna B., Duncan G.W., Khoo T.K., Yarnall A.J., Burn D.J., Rochester L. (2017). Gait Rather Than Cognition Predicts Decline in Specific Cognitive Domains in Early Parkinson’s Disease. J. Gerontol. A Biol. Sci. Med Sci..

[32] Lord S., Galna B., Verghese J., Coleman S., Burn D., Rochester L. (2013). Independent domains of gait in older adults and associated motor and nonmotor attributes: Validation of a factor analysis approach. J. Gerontol. A Biol. Sci. Med Sci..

[33] Plotnik M., Hausdorff J.M. (2008). The role of gait rhythmicity and bilateral coordination of stepping in the pathophysiology of freezing of gait in Parkinson’s disease. Mov. Disord..

[34] Santanna A., Salarian A., Wickstrom N. (2011). A new measure of movement symmetry in early parkinsons disease patients using symbolic processing of inertial sensor data. IEEE Trans. Biomed. Eng..

[35] Lord S., Galna B., Rochester L. (2013). Moving forward on gait measurement: Toward a more refined approach. Mov. Disord..

[36] Lord S., Galna B., Coleman S., Yarnall A., Burn D., Rochester L. (2014). Cognition and gait show a selective pattern of association dominated by phenotype in incident Parkinson’s disease. Front. Aging Neurosci..

[37] Lord S., Baker K., Nieuwboer A., Burn D., Rochester L. (2011). Gait variability in Parkinson’s disease: An indicator of non-dopaminergic contributors to gait dysfunction?. J. Neurol..

[38] Kroneberg D., Elshehabi M., Meyer A.-C., Otte K., Doss S., Paul F., Nussbaum S., Berg D., Kühn A.A., Maetzler W. (2019). Less is more—Estimation of the number of strides required to assess gait variability in spatially confined settings. Front. Aging Neurosci..

[39] Jacobs J.V., Earhart G.M., McNeely M.E. (2016). Can postural instability tests improve the prediction of future falls in people with Parkinson’s disease beyond knowing existing fall history?. J. Neurol..

[40] Ziegler K., Schroeteler F., Ceballos-Baumann A.O., Fietzek U.M. (2010). A new rating instrument to assess festination and freezing gait in Parkinsonian patients. Mov. Disord..

[41] Nieuwboer A., Giladi N. (2013). Characterizing freezing of gait in Parkinson’s disease: Models of an episodic phenomenon. Mov. Disord..

[42] Pintér D., Martinez-Martin P., Janszky J., Kovács N. (2019). The Parkinson’s Disease Composite Scale Is Adequately Responsive to Acute Levodopa Challenge. Parkinsons Dis..

[43] Horváth K., Aschermann Z., Acs P., Deli G., Janszky J., Komoly S., Balázs É., Takacs K., Karádi K., Kovács N. (2015). Minimal clinically important difference on the Motor Examination part of MDS-UPDRS. Parkinsonism Relat. Disord..

[44] Yogev G., Plotnik M., Peretz C., Giladi N., Hausdorff J.M. (2007). Gait asymmetry in patients with Parkinson’s disease and elderly fallers: When does the bilateral coordination of gait require attention?. Exp. Brain Res..

